# BIO (6-bromoindirubin-3′-oxime) GSK3 inhibitor induces dopaminergic differentiation of human immortalized RenVm cells

**DOI:** 10.1007/s00580-018-2696-3

**Published:** 2018-03-19

**Authors:** Mitra Soleimani, Nazem Ghasemi, Fatemeh Mohammadi Chamnari

**Affiliations:** 1 Department of Anatomical Science and Molecular Biology, School of Medicine, Isfahan University of Medical Sciences, Isfahan, Iran; 2 Faculty of Paramedicine, Isfahan University of Medical Sciences, Isfahan, Iran

**Keywords:** 6-Bromoindirubin-3′-oxime, Tyrosine hydroxylase, Beta catenin, Wnt signaling pathway

## Abstract

Parkinson’s disease (PD) is one of the most neurodegenerative disorders which can lead to severe neural disability and neurological defects. Cell-based therapy using fully differentiated cells is a new method for the treatment of this abnormal condition. In the present study, we investigated the effects of 6-bromoindirubin-3′-oxime (BIO) on dopaminergic differentiation of human immortalized RenVm cells in order to obtain a set of fully differentiated cells for transplantation in Parkinson’s disease. To this end, the immortalized RenVm cells were induced to dopaminergic differentiation using a neuro basal medium supplemented with N2 and different concentrations (75, 150, 300, 600, and 1200 nM) of BIO for 4, 8, and 12 days. The efficiency of dopaminergic differentiation was determined using immunocytochemistry for tyrosine hydroxylase expressions. In addition, the expression of a β-catenin marker was measured using the western blot technique. The results of immunocytochemistry revealed that the mean percentage of Tuj1- and TH-positive sells in 150- and 300-nM-BIO-treated groups was significantly increased compared to that of other groups (*p* ≤ 0.01). In addition, the expression of the β-catenin marker was higher in these groups as compared with that of other groups. Overall, BIO through its effect on the Wnt-Frizzled signaling pathway can promote dopaminergic differentiation of RenVm cells in a dose-dependent manner.

## Introduction

Parkinson’s disease (PD), with a prevalence of 1%, usually is detected above the age of 60. During this abnormal condition, the loss of midbrain dopamine (DA) neurons can lead to severe neural disability and neurological defects. The conventional treatment for PD is usually based on the use of immunomodulation/anti-inflammatory agents or replacement neurotransmitter (Wahner et al. 2007; Seedat et al. 2000). Although this treatment may partially reverse neuronal disturbances, it has many side effects. Thus, stem cell-based therapy may be a more effective treatment for PD (Weiss et al. 2006) and other neurodegenerative diseases (Ghasemi et al. 2014; Bantubungi et al. 2008; Blurton-Jones et al. 2009). Albeit the main mechanism responsible for this therapeutic effect is not exactly known, it seems to be due to neurotrophic effects and the differentiation potential of stem cells into functional neurons (Ghasemi et al. 2014; Park et al. 2008).

In spite of this beneficial potential of stem cell therapy, the serious adverse events of this manner such as tumorigenic potential cannot be denied. The differentiation degree of the transplanted cells is one of the most important factors which are involved in tumorigenesis. So, the molecular mechanism which is involved in signaling cell differentiation was studied by many researchers and the canonical Wnt/β-catenin pathway has been introduced as one of the important mechanisms.

Wnt proteins or cysteine-rich glycosylated proteins are the main factors which trigger the canonical Wnt/β-catenin pathway and thus regulate various cellular processes such as cell proliferation and differentiation (Nelson and Nusse 2004; Patapoutian and Reichardt 2000; Chong and Maiese 2004).

Wnt proteins consist of several members which include Wnt1, Wnt2, Wnt3, and Wnt8 proteins (Du et al. 1995). The biological activity of these proteins is mediated through the frizzled transmembrane receptor and lipoprotein-related protein 5 and 6 (LRP-5/6) (Hsieh 2004). It is important to know that the canonical Wnt/β-catenin pathway is another intracellular pathway which is activated when Wnt1 proteins bind with their receptors.

As a result, the inhibition of the downstream glycogen synthase kinase-3β (GSK-3β) occurs and β-catenin phosphorylation, ubiquitination, and subsequent degradation thorough proteosomes will be suppressed. With the increase of the cytoplasmic level of β-catenin, this factor can translocate into the cell nucleus and physically binds with DNA in order to trigger gene transcription. Overall, many special biological functions such as cellular differentiation and nervous tissue development can occur through the canonical Wnt-Frizzled signaling pathway (Kiecker and Niehrs 2001; Michiue et al. 2004; Chizhikov and Millen 2005; Panhuysen et al. 2004). Therefore, the induction of cell differentiation is possible using specific factors with potential to trigger the Wnt-Frizzled signaling pathway.

BIO (6-bromoindirubin-3′-oxime) is an agent which is used in research as an ATP-competitive inhibitor for GSK-3α/β in the Wnt signaling pathway. Previous studies suggest that this chemical compound may be a regulating target of drug resistance in colon cancer (Liu et al. 2015) and may have practical applications in regenerative medicine (Sato et al. 2004).

The immortalized cell line is undifferentiated cells that are recognized by their high self-renewal capability and multi-potency. Therefore, these cells are the main tool for research in cell-based therapy because they are capable of growing in vitro for long periods and differentiate into other cells including neurons and glial cells. The RenVM cell line is one of the immortalized human neural stem cell lines which are isolated from a 10-week-old fetal neural ventral mesencephalon and was established with the v-myc oncogene by retroviral transduction.

With respect to the broad beneficial effects of BIO in signaling cell pathways, in the current study, we evaluated the effects of several doses of BIO on dopaminergic differentiation of human immortalized RenVm cells.

## Materials and methods

### RenVm cell culture

All chemicals used in this study were purchased from Sigma-Aldrich, St. Louis, MO, USA. Moreover, the human immortalized RenVm cell lines were purchased from the Regeneron company. The immortalized RenVm cells were cultured and passaged in the pre-differentiation medium consisting of Dulbecco’s modified Eagle’s medium (DMEM) supplemented with B27, gentamicin 50 ng/ml, bFGF 10 ng/ml, EGF 20 ng/ml, and heparin 10 U/ml in a 37 °C humidified incubator with a 5% CO_2_ environment. After 80% confluency, these cells were passaged and then seeded at approximately 10,000 cells/CM2 in laminin-coated 96 wells.

### Dopaminergic differentiation of RenVm cells

The differentiation stage of RenVm cells was initiated after removal of the growth factors. For this purpose, neuro basal medium supplemented with N2, gentamicin, and different concentrations (75, 150, 300, 600, and 1200 nM) of BIO was used for 4, 8, and 12 days. The media change every other day with the ratio of 80%. The cells fixed at days 4, 8, and 12 for further experiments.

### Immunocytochemistry

After neural induction, the cells were fixed with 4% paraformaldehyde and then were permeabilized with 10% *v*/*v* normal donkey serum in PBS-Triton 0.3% *v*/*v* for 1 h at room temperature. After being washed with PBS, the cells were incubated with primary antibodies (mouse anti-TH, 1:500 dilution; anti-TuJ1, 1:1000 dilutions) overnight at 37 °C. After being washed with PBS, the cells were exposed for 1 h to 1:100 dilution of Alexa 488-conjugated goat anti-mouse IgG secondary antibody. Finally, cell nuclei were then stained with 4′,6-diamidino-2-phenylindole, dilactate (DAPI) and were observed using a fluorescence microscope (Nikon Inc., Melville, NY). For quantitative analysis, the numbers of TH- and TuJ1-positive cells were counted on each acquired image in a minimum total of 200 cells per slide, and finally, their percentage was reported.

### Western blot

Differentiated cells were subjected to western blot analysis. Briefly, the samples were lysed using lysis buffer and centrifuged at 14,000*g* for 30 min at 4 °C and the supernatant was used for immunodetection with anti β-catenin (0.25 μg/ml). Finally, appropriate secondary antibodies which conjugated with alkaline phosphatase were used and the relative expression levels of β-catenin proteins were assessed.

## Results

### RenVm cell characterization before and after differentiation

**Fig. 1:**
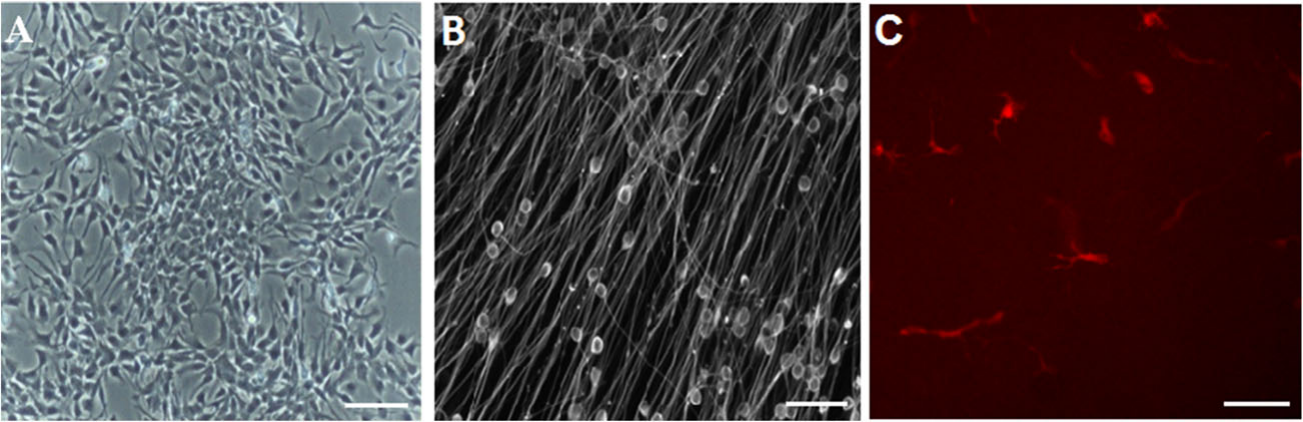
Generation of dopaminergic neuron from induced RenVm cells by different concentrations of BIO. RenVm cells at the beginning of the neural differentiation (**a**). RenVm cells which expressed Tuj1 (**b**) and tyrosine hydroxylase (TH) (**c**) markers after differentiation. Scale bars represent 200 μm in **a** and 100 μm in **b** and **c**

The assessment of RenVm cell morphology revealed that mitogenic factors (EGF and FGF-2) which are used for cell expansion can have effects on cell morphology. Before induction of cell differentiation, cultured cells expanded as islands of cells and appeared to have bipolar cell morphology (Fig. 1a). After differentiation, Re N cell VM cells formed a dense cellular network and more complex cell connections (Fig. 1b).

### Immunocytochemistry study of dopaminergic differentiation

**Fig. 2:**
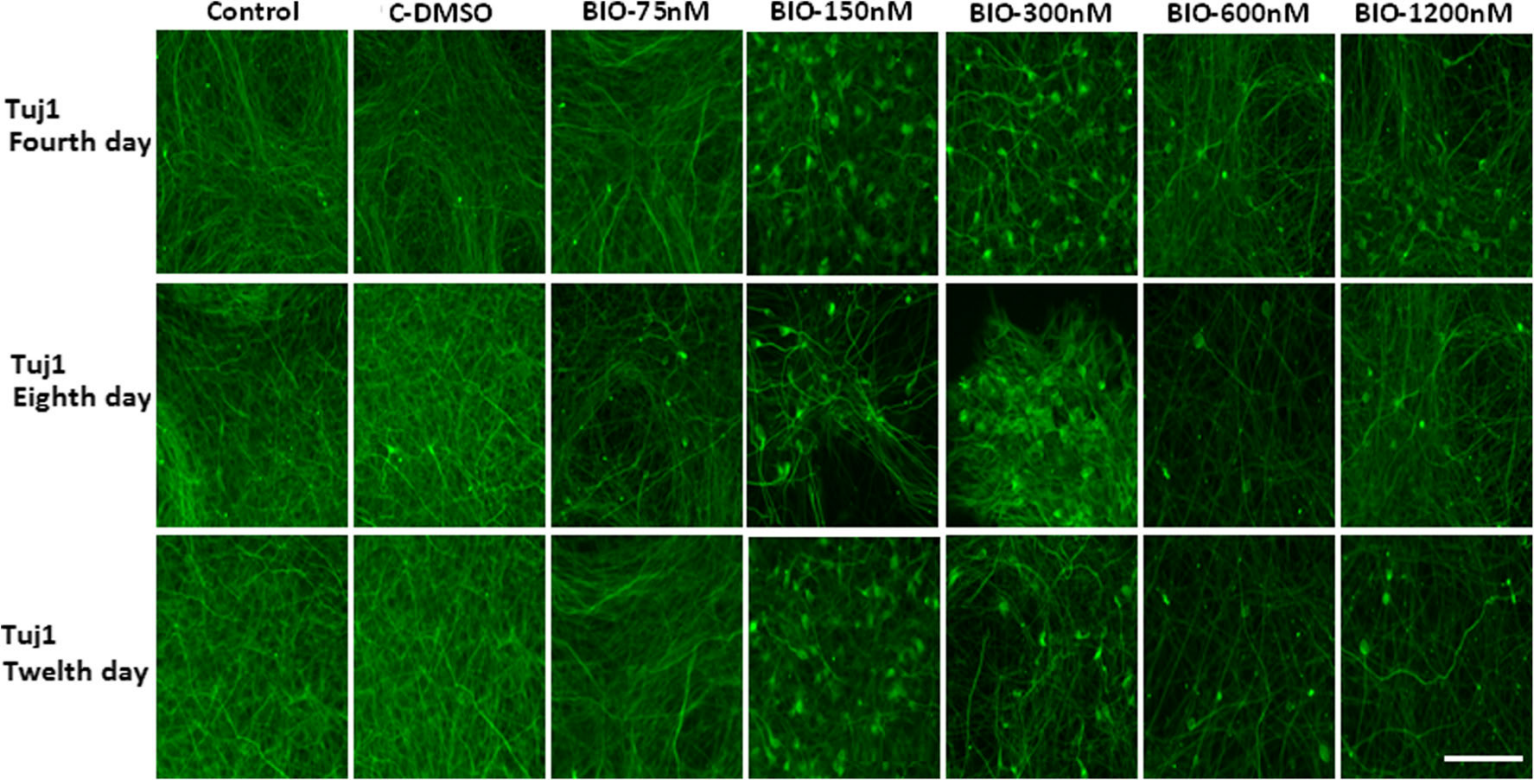
Immunocytochemistry images of differentiated cells which expressed the Tuj1 marker in different BIO concentrations. The scale bar represents 100 μm

**Fig. 3:**
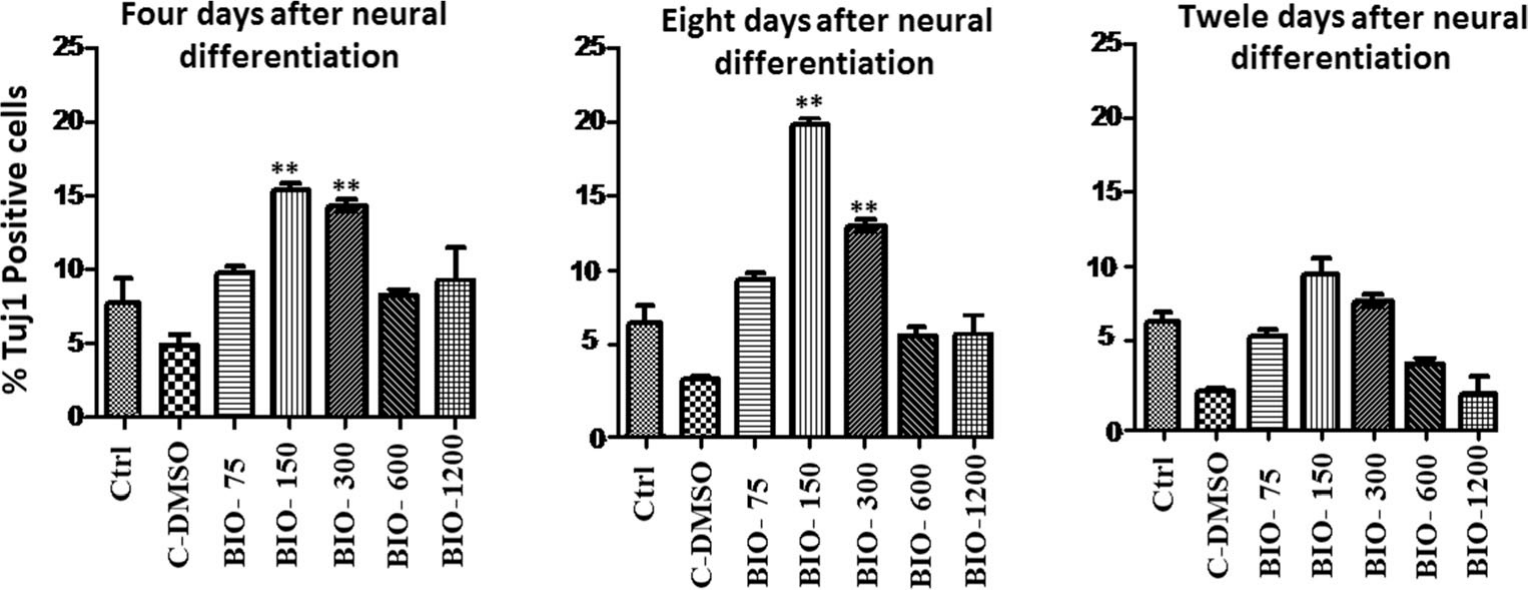
The mean percentage of differentiated cells which expressed the Tuj1 markers. In the 150 and 300 nM concentrations of BIO in the fourth and eighth days, the mean percentage of Tuj1-positive cells was significantly increased compared to that of the other groups (**p ≤ 0.01)

**Fig. 4:**
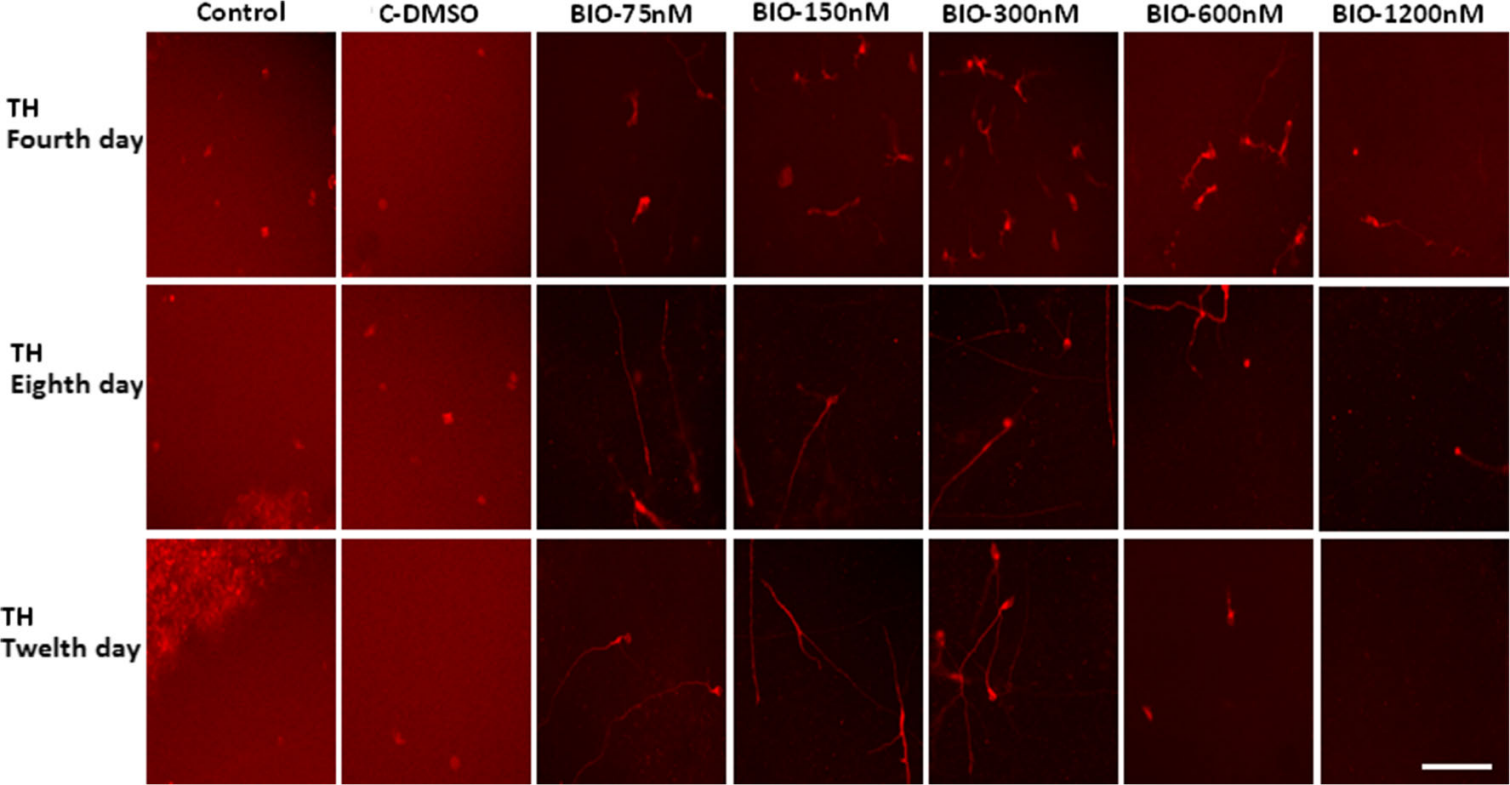
Immunocytochemistry images of differentiated cells which expressed the tyrosine hydroxylase (TH) marker in different BIO concentrations. The scale bar represents 100 μm

**Fig. 5:**
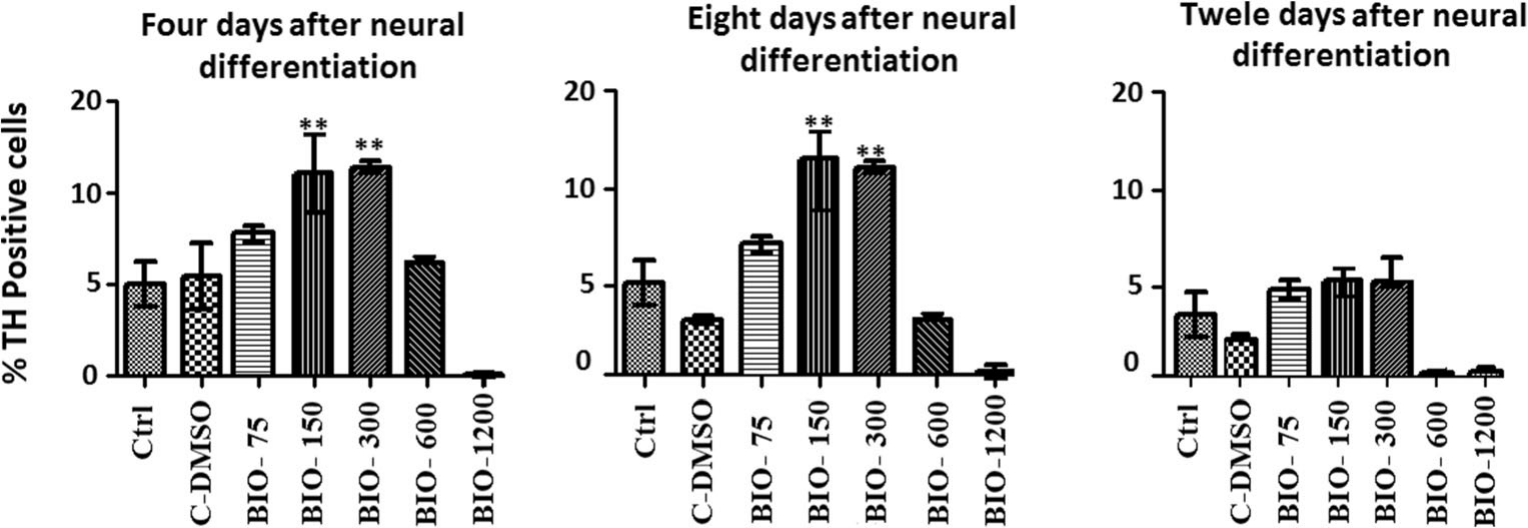
The mean percentage of differentiated cells which expressed the tyrosine hydroxylase (TH) marker. In the 150 and 300 nM concentrations of BIO in the fourth and eighth days, the mean percentage of TH-positive cells was significantly increased compared to that of the other groups (**p ≤ 0.01)

Fluorescence microscopic analysis with specific markers revealed that the mean percentage of cells which expressed Tuj1 and TH markers was higher in the presence of 150 and 300 nM of BIO on the fourth and seventh days when compared with that of other groups (*p* ≤ 0.01) (Figs. 2, 3, 4, and 5).

### Western blot analysis

**Fig. 6:**
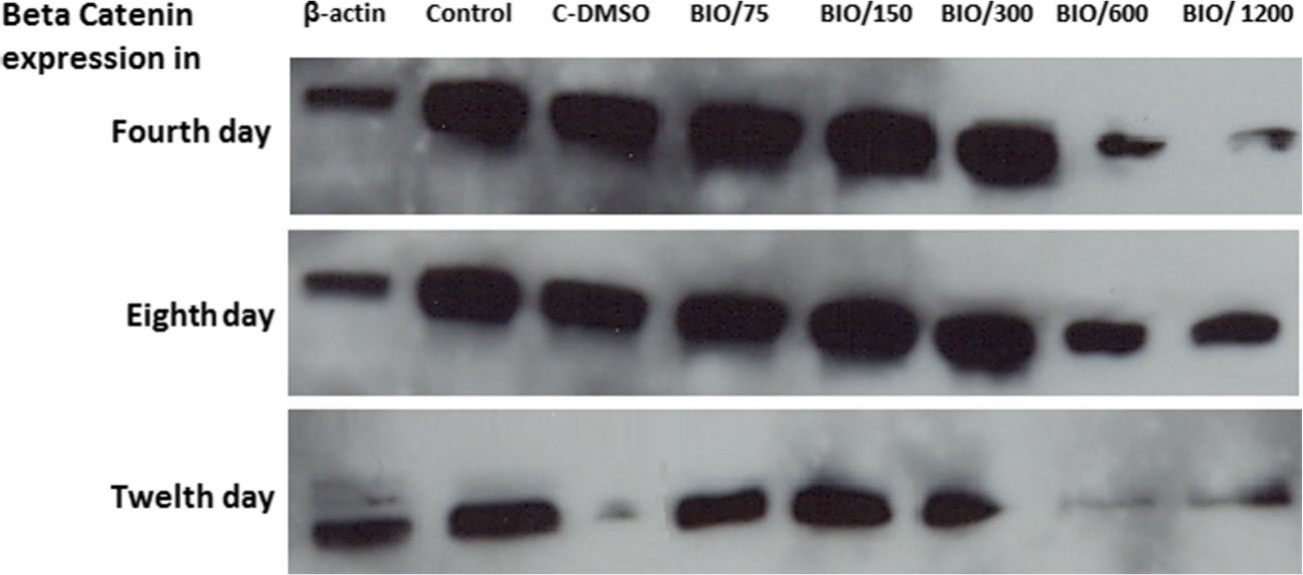
Western analysis of β-catenin in differentiated and undifferentiated cells in different concentrations of BIO at 4, 8, and 12 days. β-Actin was used as a control marker

After neural induction, protein was extracted from differentiated cells for western blot assays. To this end, β-actin was used as a control marker. Our results demonstrated that the expression of β-catenin markers was higher in differentiated cells which were induced with 150 and 300 nM concentrations of BIO compared with that of the other groups especially in the fourth and eighth days which is consistent with the results of immunohistochemistry (Fig. 6).

## Discussion

Stem cell therapy is a new strategy for the treatment of neurodegenerative diseases such as Parkinson’s (Weiss et al. 2006) and usually is done with the aim of replacing the damaged cells. The choice of a suitable cell source for this strategy is one of the most important points that should be noted. Undifferentiated cell transplantation can lead to serious complications such as the emergence of tumors in non-damaged areas (Amariglio et al. 2009). Thus, there is much concern about the safety of this therapy. The transplantation of fully differentiated cells instead of stem cells can play a role in reducing these adverse events. To this end, the present study was done in order to promote dopaminergic neuron differentiation by BIO in order to access a valuable population of differentiated cells for cell transplantation in Parkinson’s disease.

BIO is one of the important factors that may change gene expression through the effects on cell signaling such as the canonical Wnt-Frizzled signaling pathway. This pathway is initiated by the interaction of Wnt1 proteins including Wnt1, Wnt2, Wnt3, and Wnt8 with Frizzled receptors in the presence of the co-receptor LRP-5/6 (Mao et al. 2001; Pinson et al. 2000; Wehrli et al. 2000). Thus, a tri-molecular complex namely Wnt-Frizzled-LRP5/6 complex is formed. In the following, this complex by recruitment of a cytoplasmic phosphoprotein (Disheveled) is able to inhibit the GSK-3β activity (Kishida et al. 2001; Fukumoto et al. 2001). As a result, β-catenin which is associated with the GSK-3β will not be phosphorylated. So, β-catenin by connecting to the nucleus is able to activate the transcription of specific genes which are involved in cell proliferation and differentiation.

Previous studies suggest that many cytokines and growth factors such as fibroblast growth factor 2 via inhibiting the GSK-3 activity and raising the nuclear levels of β-catenin are able to promote angiogenesis (Dono et al. 2002; Holnthoner et al. 2002). In addition, it has been reported that the loss of Wnt signaling or mutation in a member of the Wnt-Frizzled-LRP5/6 complex is able to induce neurodegeneration (Jones et al. 2000). As shown in Fig. 4, the induction of the TH expression in RenVm cells was dose-dependent. Additionally, western blot results demonstrated that the β-catenin expression level also has increased in the groups which were treated with BIO (especially in the 150 and 300 nM concentrations) compared with the control and DMSO groups. Thus, it can be justified that BIO may participate in promoting dopaminergic differentiation by inhibition of the GSK-3β activity through inhibition of β-catenin phosphorylation. Therefore, β-catenin can enter the nucleus and, by binding to DNA, is able to increase the transcription of specific genes which are involved in dopaminergic differentiation.

## Conclusion

It can be concluded that BIO is able to inhibit both GSK-3β activity and β-catenin phosphorylation by its effect on the Wnt-Frizzled signaling pathway. Thus, this agent may facilitate differentiation of RenVm cells to dopaminergic neuronal cells.
